# Managing the painful blind eye

**Published:** 2022-01-31

**Authors:** Jason A Penniecook, Doris Saraí Garza Cruz, Maria Soledad Pighin Caminos

**Affiliations:** 1 Ophthalmologist (Glaucoma): Instituto de la Visión, Centro Mexicano de Salud Visual Preventiva, Collaborative Network for Quality in Eye Research, Montemorelos, Mexico.; 2 Ophthalmologist (Anterior Segment): Instituto de la Visión, Montemorelos/Merida, Mexico.; 3 Ophthalmologist (Retina): Institut Català de Retina, Barcelona, Spain.

**Sometimes, despite all efforts to help a patient, visual function is lost permanently and the eye undergoes degenerative changes that can lead to persistent pain. Understanding that vision cannot be restored will help patients to accept suitable treatment.**

**Figure 1:**
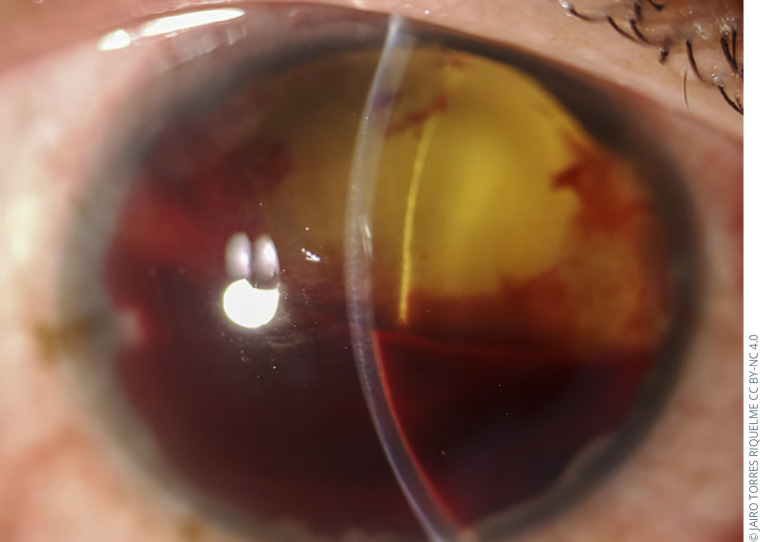
Neovascular glaucoma often results in a painful blind eye. This eye has a collapsed anterior chamber with angle closure resulting from neovascular glaucoma secondary to proliferative diabetic retinopathy.

Although definitions vary widely, a working definition for a painful blind eye is one with a visual acuity of counting fingers or worse that has no realistic probability of recovering function and is accompanied by ongoing chronic pain and ongoing discomfort which has lasted for at least four weeks.

## Causes of the painful, blind eye

A painful, blind eye may result from any disease that causes blindness or a phthisical (shrunken, scarred, and non-functioning) eye. Acute causes include chemical or physical trauma, and chronic conditions include corneal decompensation and advanced and intractable glaucoma, especially neovascular glaucoma.

## Understanding eye pain

Mechanical, temperature, irritant and inflammation stimuli in the eye are detected by specialised nerve terminals of the trigeminal nerve (called nociceptors) which send signals to the brain in response to injury or damage in the eye tissues. These nerve terminals are part of the **peripheral nervous system**, whereas the brain and spinal cord form part of the **central nervous system**.

The signals are interpreted by the brain as different levels of discomfort, including pain, burning, or stinging, and the response can include increased tearing, blinking, protective movements, and verbal expressions.

There are two types of eye pain:

- **Physiological (normal) pain** resulting from damage to eye tissues. Inflammation (e.g., from uveitis) can also cause increased sensitisation of the nociceptors, resulting in persistent pain that is exaggerated in comparison to any tissue damage.^1^^,^^2^
- **Neuropathic pain** resulting from damage to the nociceptors or the other structures involved in detecting, transmitting and processing pain signals between the peripheral nervous system and the brain. This abnormal signalling response can cause sensations of discomfort and pain in response to non-painful stimuli.^1^^,^^2^

## How to manage a painful, blind eye

There are very few evidence-based approaches for managing the painful blind eye. Caregivers should try to differentiate between **physiological pain** and **neuropathic pain**, but this can be challenging.

**Physiological pain.** Pain that responds to anaesthetic eye drops, such as proparacaine hydrochloride, or steroid drops, implies that this is physiological pain originating in the peripheral nerves. Physiological pain can be reduced by treating the underlying cause of the pain and/or by reducing inflammation.

**Neuropathic pain.** Eyes that don’t respond to anaesthetic drops, or steroid drops in the case of ocular inflammation, suggest a neuropathic type of pain which can be very difficult to treat. Systemic, or even psychological, interventions may be required (ideally via a specialised pain management clinic, although these are not widely available).

Whatever the cause of the pain, the most important management aim is to reduce, or help the patient cope with, the pain. Possible methods of management are outlined below. Alternatives that can be safely performed in the clinical office without the need for a surgical room are classified as “non-invasive” in this article.

Treatment of the underlying cause is important, it is also essential that the patient understands the irreversible loss of visual function; this will help when discussing some of the management options, particularly the more invasive types of treatment.

### Non-invasive treatments

The first line of treatment usually consists of topical eye drops that may include lubricants, anti-inflammatory drugs (both steroidal and non-steroidal) as well as immunomodulators.

Eye drops containing **atropine** (a cycloplegic) are commonly used to reduce possible **ciliary spasm**.

A combination of **pressure-lowering, steroid, and cycloplegic drops used in the long term** can help to reduce pain for patients with a painful blind eye resulting from **neovascular glaucoma.**

**Steroid drops**, **lubricants** and, in some cases, **therapeutic contact lenses** are useful for patients with **corneal issues**, for example, a painful blind eye after multiple failed corneal transplants.

**Retrobulbar injection of absolute (100%) alcohol** to destroy the sensory ciliary nerves has also been used as a pain management option.^2^

**Systemic drugs**, including **antidepressants** and **anticonvulsants**, can be used for some types of neuropathic corneal pain. These would be given in collaboration with a family doctor or at a pain clinic.

**Figure 2:**
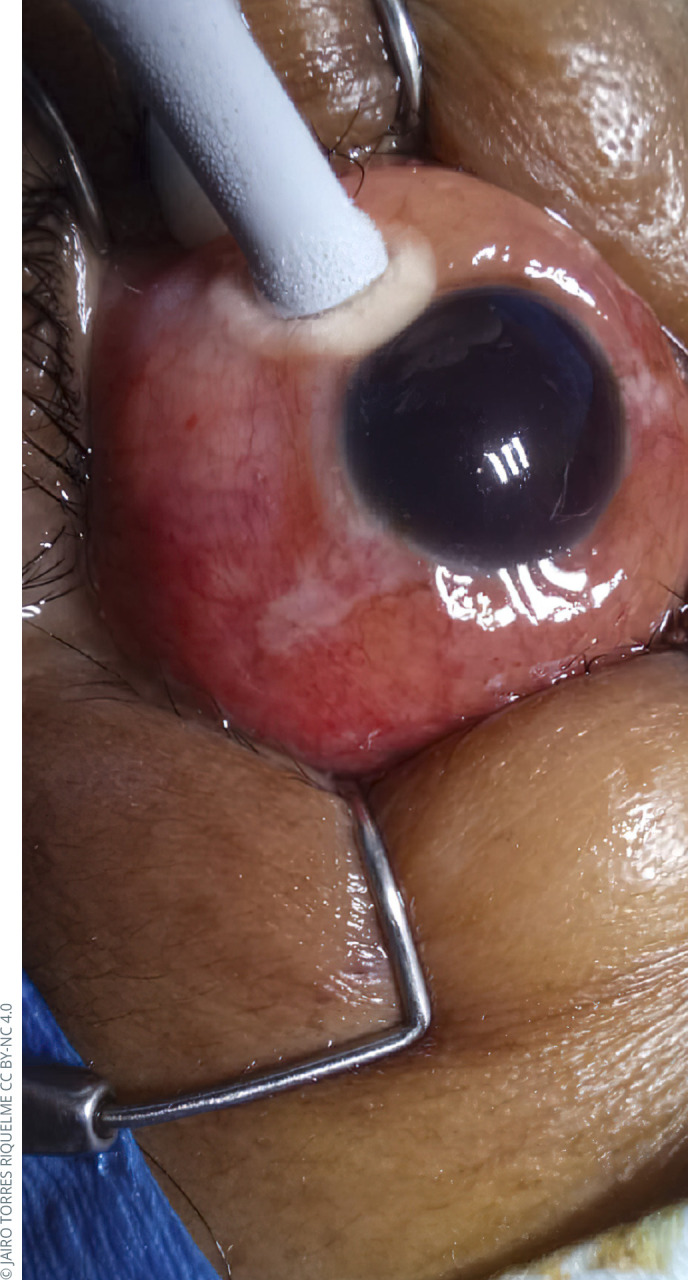
Cyclocryotherapy to reduce pressure and relieve pain in a painful blind eye.

### Invasive treatment

Where a **high intraocular pressure** is likely contributing to the pain, **transscleral and endocyclophotocoagulation, high-intensity focused ultrasound (ultrasound cycloplasty)**,^3^ and **cyclocryotherapy**^3^ can be used. Destruction of the ciliary body and reduced aqueous production is the common mechanism of action and they offer the advantage of repeated treatments. Cyclocryotherapy is appropriate where vision has been lost completely.

Where the cause of the pain is a **damaged or non-healing corneal surface**, procedures such as an amniotic membrane graft or conjunctival advancement (Gunderson) flap will bring relief.

**Surgical correction** and even **implantation of electrode devices** have been used for **neuropathic ocular surface-related disorders**.

**Evisceration** (removal of the eyeball contents while leaving the sclera, the outer layer of the eyeball behind) and **enucleation** (complete removal of the eyeball) are the final alternatives that can grant definitive relief.^4^

## Conclusion

An understanding of the underlying mechanism, and awareness of the different treatment options, will help direct the best approach for individual patients and achieve sustained pain relief.

**Table 1:** Summary

**Common clinical presentations** Following trauma (e.g., mechanical, chemical, thermal) Severe glaucoma, especially neovascular glaucoma (common causes include diabetic retinopathy and retinal vein occlusion) Endophthalmitis Microbial keratitis Uveitis
**Non-invasive treatment options** Drops to lower the intraocular pressure (if it is elevated) Steroid eye drops Oral analgesics including NSAIDs or paracetamol Therapeutic (bandage) contact lens for corneal surface damage Retrobulbar injection of alcohol or chlorpromazine Oral neuropathic pain medication (e.g., amitriptyline or gabapentin)
**Invasive treatment options** To lower an elevated intraocular pressure: Transcleral or endocyclophotocoagulation Ultrasonic cycloplasty Cyclocryotherapy (when vision has been lost completely) Corneal protection surgery Amniotic membrane graft Conjunctival advancement flap As a last resort: eye removal Evisceration Enucleation
